# Supplementary material for: Conservation and divergence of sex-biased gene expression across 50 million years of Drosophila evolution
Source: Mol Biol Evol. 2026 Apr 6;43(4):msag081. doi: 10.1093/molbev/msag081 (PMC13107567; doi:10.1093/molbev/msag081)
Supplement: msag081_Supplementary_Data [file msag081_supplementary_data.zip › SuppFigs.pdf]

Supplementary Figures

Conservation and divergence of sex-biased gene expression across 50 million years of *Drosophila* evolution

Amanda Glaser-Schmitt* and John Parsch*

Division of Evolutionary Biology, Faculty of Biology, Ludwig-Maximilians-Universität München, Munich, Germany

*Corresponding authors:

Email: glaser@bio.lmu.de (AGS); parsch@bio.lmu.de (JP)

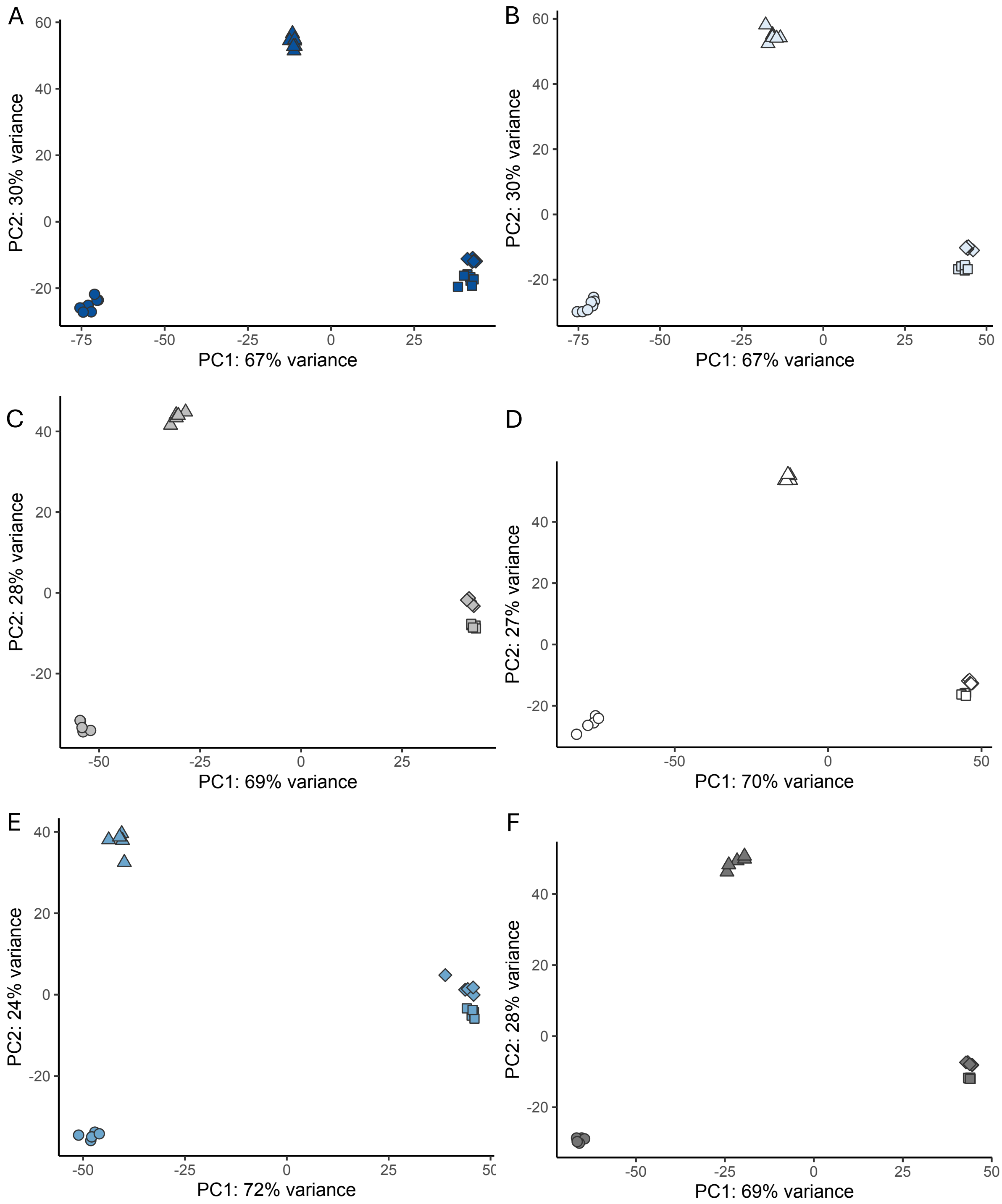

Fig S1: Principal component analysis of gene expression profiles in A) *Dmel*, B) *Dsim*, C) *Dsuz*, D) *Dana*, E) *Dsub*, and F) *Dimm*. Triangles and diamonds indicate female and male body, respectively while circles and squares indicate female and male head, respectively.

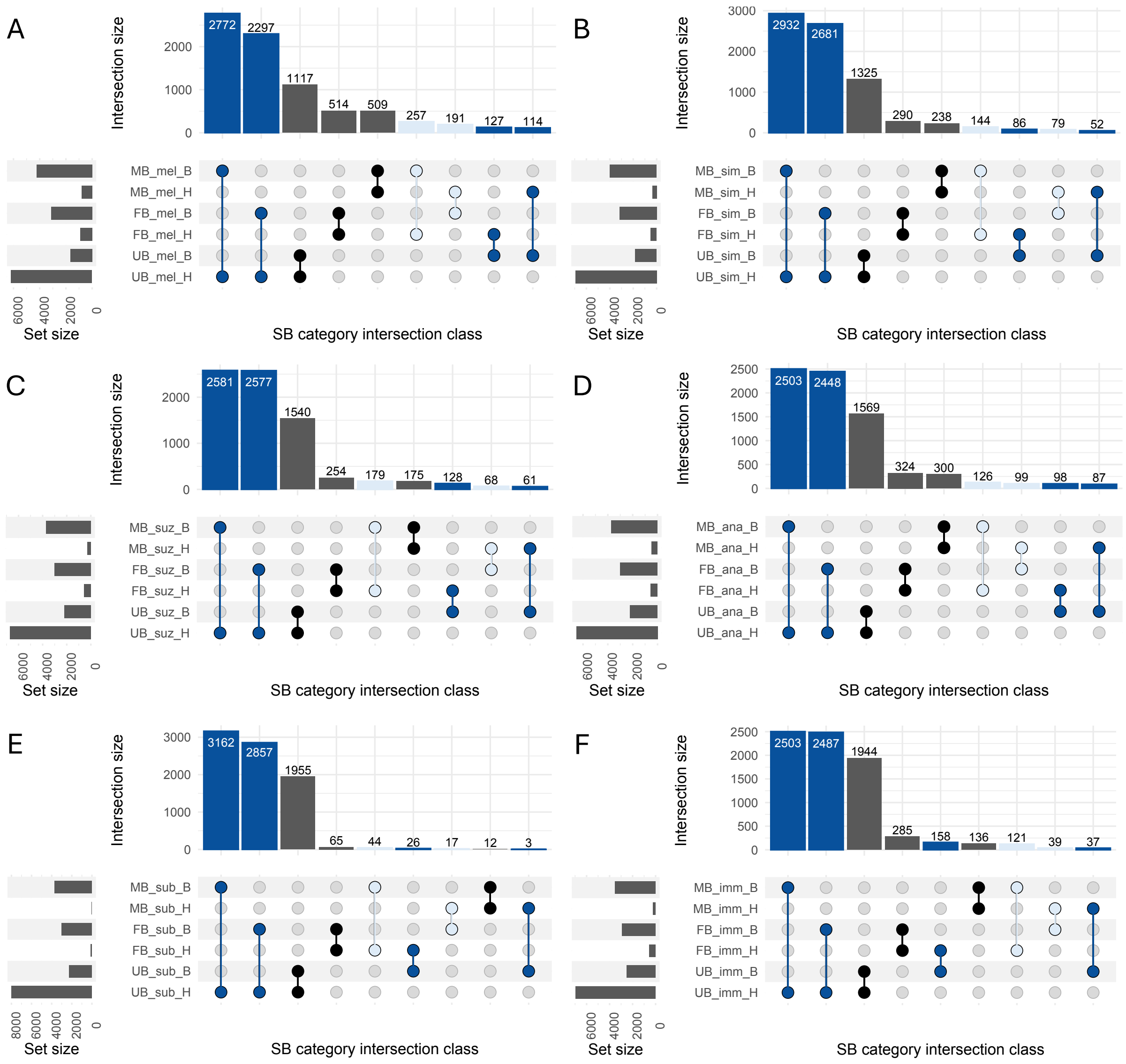

Fig S2: Overlap in SB genes between head (H) and body (B). Shown are upset plots demonstrating the overlap in UB, FB, or MB genes in A) *Dmel* (mel), B) *Dsim* (sim), C) *Dsuz* (suz), D) *Dana* (ana), E) *Dsub* (sub), and F) *Dimm* (imm). Horizontal bars represent the total number of genes in a body part and sex bias category combination. Vertical bars represent the number of genes in an intersection class. Connected, filled circles underneath a vertical bar indicate that a body part and SB category combination is included in an intersection class. Blue represents genes UB in one body part and SB in the other, dark gray represents gene in the same bias category, and light blue indicates genes with the opposite sex bias between head and body.

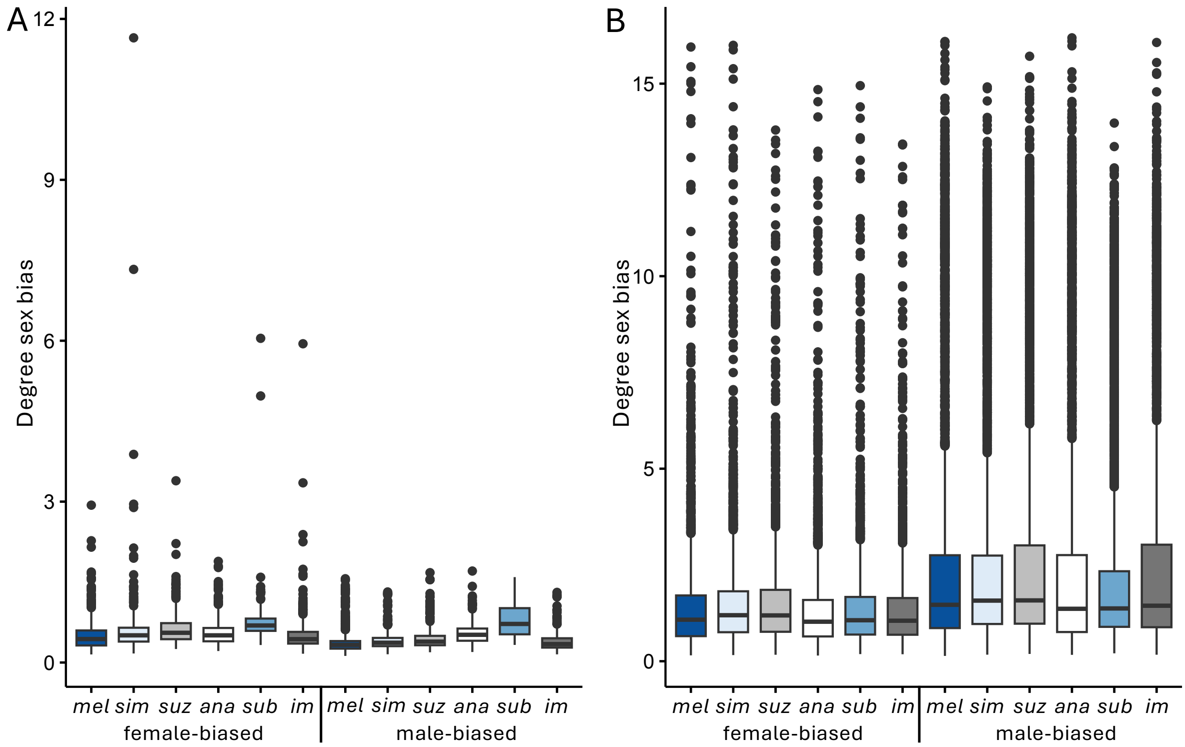

C

MB \ FB	*mel*	*sim*	*suz*	*ana*	*sub*	*imm*
mel		3.18E-03	4.89E-15	1.96E-04	6.44E-07	**1**
sim	1.65E-03		**1**	**0.657**	7.59E-03	4.12E-02
suz	9.42E-08	**0.058**		1.65E-04	5.56E-03	4.37E-07
ana	1.54E-15	1.54E-15	4.86E-06		6.01E-05	**0.212**
sub	1.06E-06	7.57E-06	5.87E-05	2.04E-03		4.18E-06
imm	**0.278**	**1**	3.01E-02	1.12E-14	4.57E-06	

D

MB \ FB	*mel*	*sim*	*suz*	*ana*	*sub*	*imm*
mel		8.58E-03	2.67E-03	**0.736**	**1**	**1**
sim	**1**		**1**	1.15E-05	1.68E-03	5.67E-05
suz	**0.340**	**0.388**		1.94E-06	4.36E-04	1.04E-05
ana	**1**	**1**	**0.774**		**1**	**1**
sub	6.95E-07	2.19E-07	1.98E-12	5.45E-07		**1**
imm	**1**	**1**	**1**	**1**	8.96E-08	

Fig S3: Magnitude of sex bias in the A) head and B) body of *Dmel* (mel), *Dsim* (sim), *Dsuz* (suz), *Dana* (ana), *Dsub* (sub), and *Dimm* (im/imm). Significant differences between species for MB and FB genes were assessed with a *t*-test. Shown are the BH-corrected *P*-values in the C) head and D) body. Non-significant *P*-values are shown in bold.

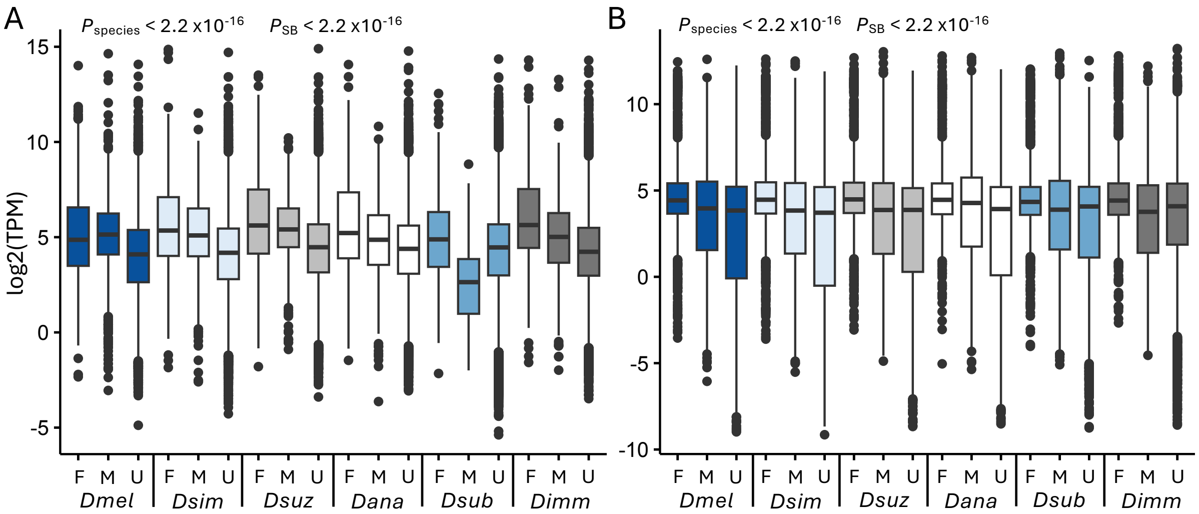

Fig S4: Overall expression levels in the A) head and B) body for MB (M), FB (F), and UB (U) genes in *Dmel*, *Dsim*, *Dsuz*, *Dana*, *Dsub*, and *Dimm*. Significance was assessed for each body part with a type II ANOVA with sex bias (SB) category and species as fixed factors and gene as a random factor.

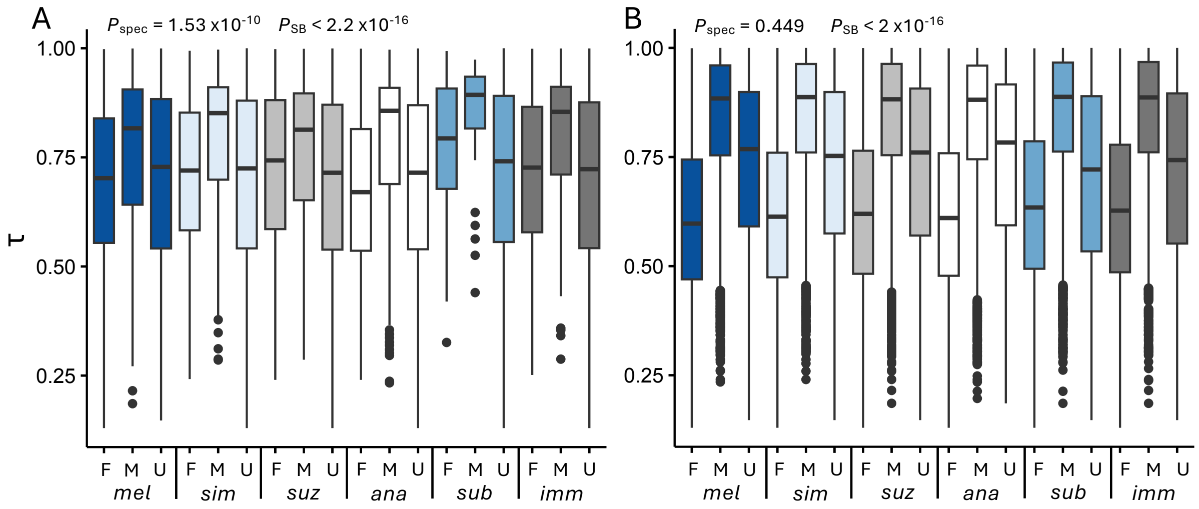

Fig S5: Tissue specificity τ in the A) head and B) body for MB (M), FB (F), and UB (U) genes in *Dmel*, *Dsim*, *Dsuz*, *Dana*, *Dsub*, and *Dimm*. Significance was assessed for each body part with a type II ANOVA with sex bias (SB) category and species as factors.

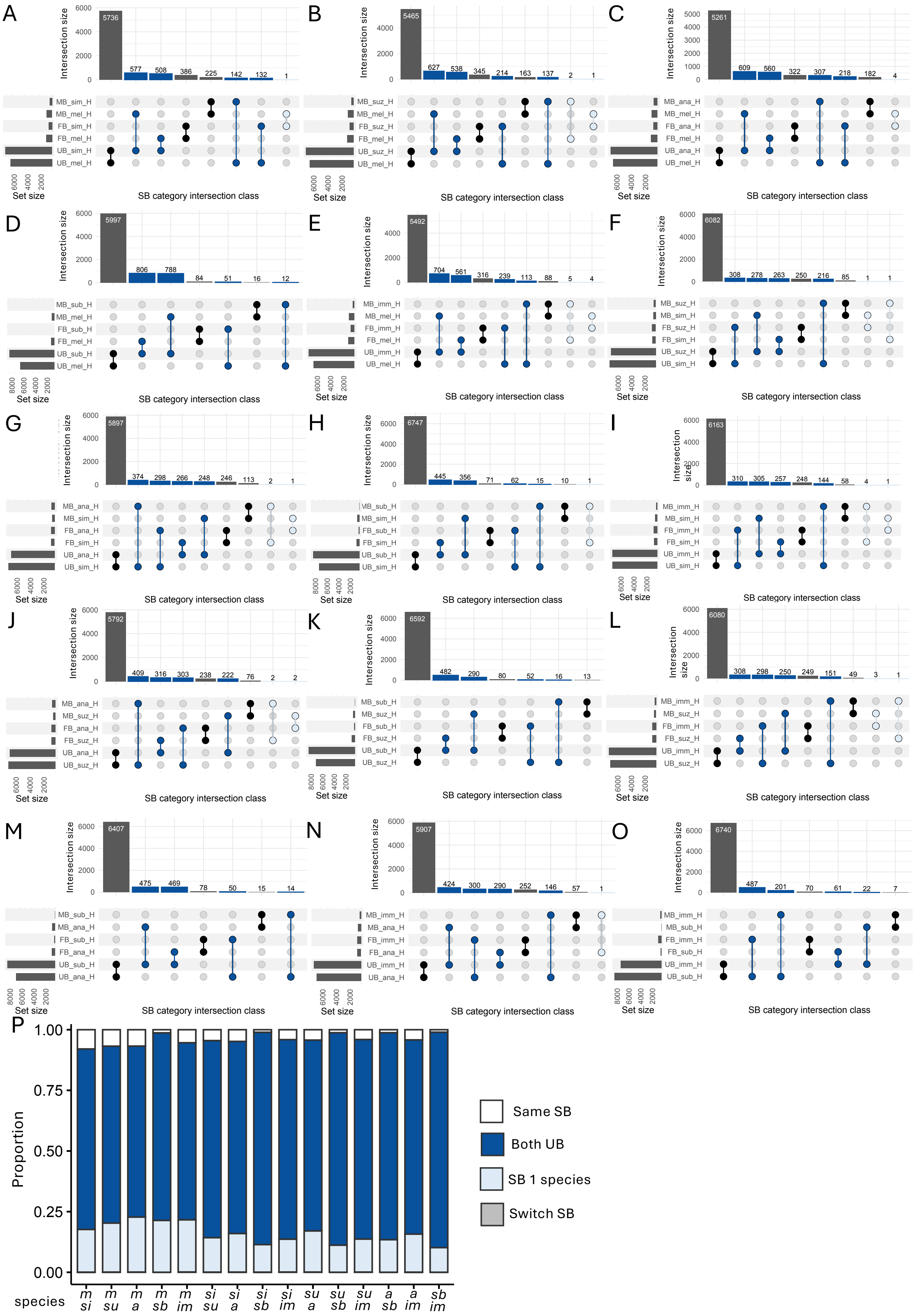
A
Intersection size
5736 577 508 386 225 142 132 1
MB_sim_H
MB_mel_H
FB_sim_H
FB_mel_H
UB_sim_H
UB_mel_H
Set size
SB category intersection class
B
Intersection size
5465 627 538 345 214 163 137 2 1
MB_suz_H
MB_mel_H
FB_suz_H
FB_mel_H
UB_suz_H
UB_mel_H
Set size
SB category intersection class
C
Intersection size
5261 609 560 322 307 218 182 4
MB_ana_H
MB_mel_H
FB_ana_H
FB_mel_H
UB_ana_H
UB_mel_H
Set size
SB category intersection class
D
Intersection size
5997 806 788 84 51 16 12
MB_sub_H
MB_mel_H
FB_sub_H
FB_mel_H
UB_sub_H
UB_mel_H
Set size
SB category intersection class
E
Intersection size
5492 704 561 316 239 113 88 5 4
MB_imm_H
MB_mel_H
FB_imm_H
FB_mel_H
UB_imm_H
UB_mel_H
Set size
SB category intersection class
F
Intersection size
6082 308 278 263 250 216 85 1 1
MB_suz_H
MB_sim_H
FB_suz_H
FB_sim_H
UB_suz_H
UB_sim_H
Set size
SB category intersection class
G
Intersection size
5897 374 298 266 248 246 113 2 1
MB_ana_H
MB_sim_H
FB_ana_H
FB_sim_H
UB_ana_H
UB_sim_H
Set size
SB category intersection class
H
Intersection size
6747 445 356 71 62 15 10 1
MB_sub_H
MB_sim_H
FB_sub_H
FB_sim_H
UB_sub_H
UB_sim_H
Set size
SB category intersection class
I
Intersection size
6163 310 305 257 248 144 58 4 1
MB_imm_H
MB_sim_H
FB_imm_H
FB_sim_H
UB_imm_H
UB_sim_H
Set size
SB category intersection class
J
Intersection size
5792 409 316 303 238 222 76 2 2
MB_ana_H
MB_suz_H
FB_ana_H
FB_suz_H
UB_ana_H
UB_suz_H
Set size
SB category intersection class
K
Intersection size
6592 482 290 80 52 16 13
MB_sub_H
MB_suz_H
FB_sub_H
FB_suz_H
UB_sub_H
UB_suz_H
Set size
SB category intersection class
L
Intersection size
6080 308 298 250 249 151 49 3 1
MB_imm_H
MB_suz_H
FB_imm_H
FB_suz_H
UB_imm_H
UB_suz_H
Set size
SB category intersection class
M
Intersection size
6407 475 469 78 50 15 14
MB_sub_H
MB_ana_H
FB_sub_H
FB_ana_H
UB_sub_H
UB_ana_H
Set size
SB category intersection class
N
Intersection size
5907 424 300 290 252 146 57 1
MB_imm_H
MB_ana_H
FB_imm_H
FB_ana_H
UB_imm_H
UB_ana_H
Set size
SB category intersection class
O
Intersection size
6740 487 201 70 61 22 7
MB_imm_H
MB_sub_H
FB_imm_H
FB_sub_H
UB_imm_H
UB_sub_H
Set size
SB category intersection class
P
Proportion
1.00
0.75
0.50
0.25
0.00
species
m si
m su
m a
m sb
m im
si su
si a
si sb
si im
su a
su sb
su im
a sb
a im
sb im
Same SB
Both UB
SB 1 species
Switch SB

Fig S6: Overlap in SB genes within the head (H) between species. Shown are upset plots demonstrating the overlap in UB, FB, or MB genes in pairwise comparisons between A–E) *Dmel* (mel), A, F–I) *Dsim* (sim), B,F,J–L) *Dsuz* (suz), C,G,J,M,N) *Dana* (ana), D,H,K,M,O) *Dsub* (sub), and E,I,L,N,O) *Dimm* (imm). Horizontal bars represent the total number of genes in a species and sex bias category combination. Vertical bars represent the number of genes in an intersection class. Connected, filled circles underneath a vertical bar indicate that a species and sex bias category combination is included in an intersection class. Blue represents genes UB in one species and SB in the other, dark gray represents genes in the same sex bias category, and light blue indicates genes that switch sex bias between species. P) Summary of pairwise overlap in sex bias categories among species. Shown are the proportion of overlapping genes that had the same SB (white) or were both UB (dark blue), were SB in only one of the examined species (SB 1 species; light blue), or switched sex bias (Switch SB; gray) between *Dmel* (*m*), *Dsim* (*si*), *Dsuz* (*su*), *Dana* (*a*), *Dsub* (*sb*), and *Dimm* (*im*).

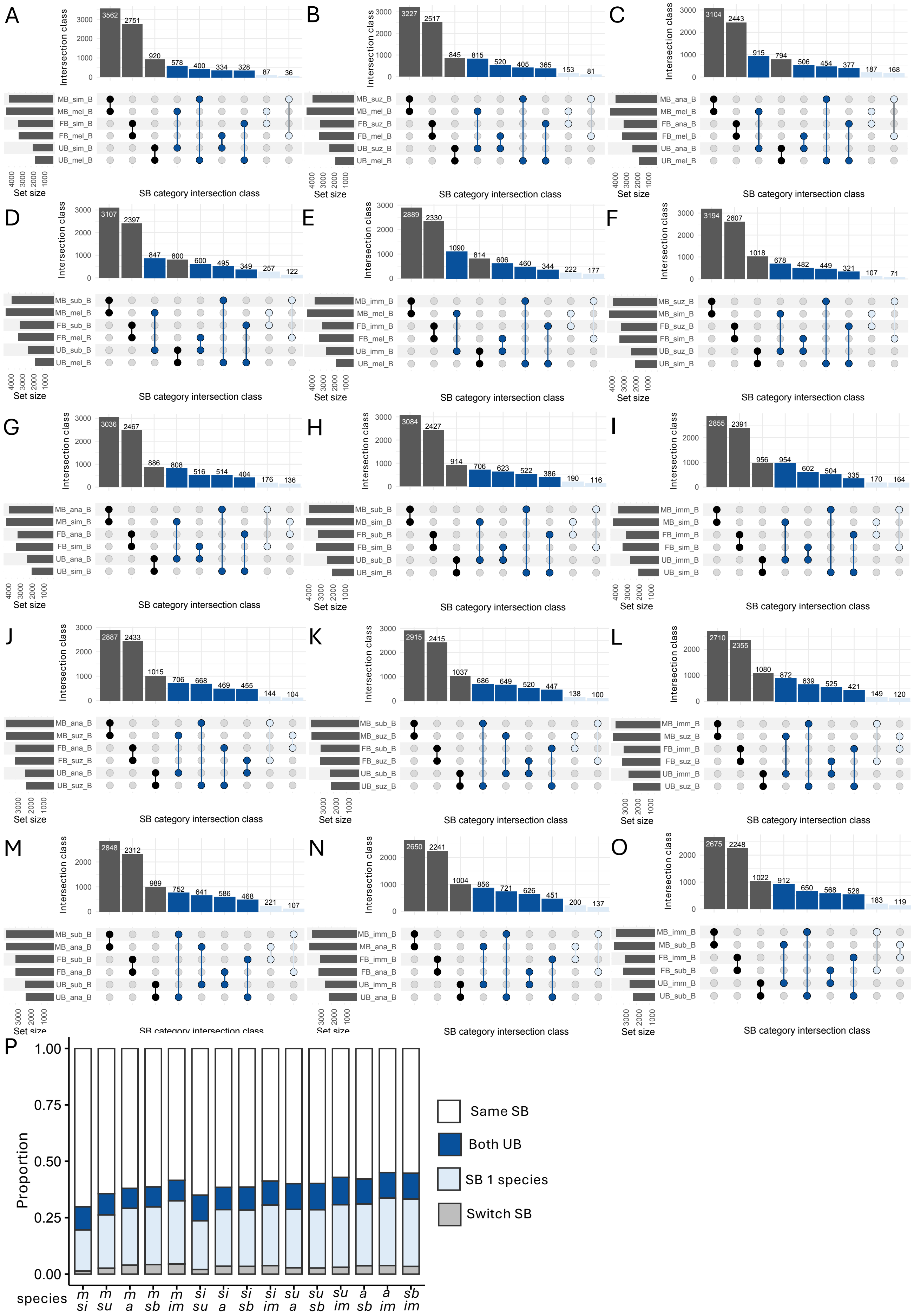
A
3562 2751 920 578 400 334 328 87 36
MB_sim_B MB_mel_B FB_sim_B FB_mel_B UB_sim_B UB_mel_B
B
3227 2517 845 815 520 405 365 153 81
MB_suz_B MB_mel_B FB_suz_B FB_mel_B UB_suz_B UB_mel_B
C
3104 2443 915 794 506 454 377 187 168
MB_ana_B MB_mel_B FB_ana_B FB_mel_B UB_ana_B UB_mel_B
D
3107 2397 847 800 600 495 349 257 122
MB_sub_B MB_mel_B FB_sub_B FB_mel_B UB_sub_B UB_mel_B
E
2889 2330 1090 814 606 460 344 222 177
MB_imm_B MB_mel_B FB_imm_B FB_mel_B UB_imm_B UB_mel_B
F
3194 2607 1018 678 482 449 321 107 71
MB_suz_B MB_sim_B FB_suz_B FB_sim_B UB_suz_B UB_sim_B
G
3036 2467 886 808 516 514 404 176 136
MB_ana_B MB_sim_B FB_ana_B FB_sim_B UB_ana_B UB_sim_B
H
3084 2427 914 706 623 522 386 190 116
MB_sub_B MB_sim_B FB_sub_B FB_sim_B UB_sub_B UB_sim_B
I
2855 2391 956 954 602 504 335 170 164
MB_imm_B MB_sim_B FB_imm_B FB_sim_B UB_imm_B UB_sim_B
J
2887 2433 1015 706 668 469 455 144 104
MB_ana_B MB_suz_B FB_ana_B FB_suz_B UB_ana_B UB_suz_B
K
2915 2415 1037 686 649 520 447 138 100
MB_sub_B MB_suz_B FB_sub_B FB_suz_B UB_sub_B UB_suz_B
L
2710 2355 1080 872 639 525 421 149 120
MB_imm_B MB_suz_B FB_imm_B FB_suz_B UB_imm_B UB_suz_B
M
2848 2312 989 752 641 586 468 221 107
MB_sub_B MB_ana_B FB_sub_B FB_ana_B UB_sub_B UB_ana_B
N
2650 2241 1004 856 721 626 451 200 137
MB_imm_B MB_ana_B FB_imm_B FB_ana_B UB_imm_B UB_ana_B
O
2675 2248 1022 912 650 568 528 183 119
MB_imm_B MB_sub_B FB_imm_B FB_sub_B UB_imm_B UB_sub_B
Intersection class
Set size
SB category intersection class
P
Proportion
1.00
0.75
0.50
0.25
0.00
species
m si
m su
m a
m sb
m im
si su
si a
si sb
si im
su a
su sb
su im
a sb
a im
sb im
Same SB
Both UB
SB 1 species
Switch SB

Fig S7: Overlap in sex biased genes within the body (B) between species. Shown are upset plots demonstrating the overlap in UB, FB, or MB genes in pairwise comparisons between A–E) *Dmel* (mel), A, F–I) *Dsim* (sim), B,F,J–L) *D. suz* (suz), C,G,J,M,N) *Dana* (ana), D,H,K,M,O) *Dsub* (sub), and E,I,L,N,O) *Dimm* (imm). Horizontal bars represent the total number of genes in a species and sex bias category combination. Vertical bars represent the number of genes in an intersection class. Connected, filled circles underneath a vertical bar indicate that a species and sex bias category combination is included in an intersection class. Blue represents genes UB in one species and SB in the other, dark gray represents genes in the same sex bias category, and light blue indicates genes that switch sex bias between species. P) Summary of pairwise overlap in sex bias categories among species. Shown are the proportion of overlapping genes that had the same SB (white) or were both UB (dark blue), were SB in only one of the examined species (SB 1 species; light blue), or switched sex bias (Switch SB; gray) between *Dmel* (*m*), *Dsim* (*si*), *Dsuz* (*su*), *Dana* (*a*), *Dsub* (*sb*), and *Dimm* (*im*).

A

i) 2 (0.019)	*ii)* 21 (0.200)
iii) 79 (0.752)	*iv)* 3 (0.029)

$Total_{con}$ = 100 (0.952)
$Total_{opp}$ = 5 (0.048)

B

i) 31 (0.207)	*ii)* 15 (0.100)
iii) 85 (0.567)	*iv)* 12 (0.127)

$Total_{con}$ = 100 (0. 667)
$Total_{opp}$ = 50 (0. 333)

C

i) 221 (0.136)	*ii)* 593 (0.365)
iii) 615 (0.378)	*iv)* 196 (0.121)

$Total_{con}$ = 1208 (0.743)
$Total_{opp}$ = 417 (0.257)

D

i) 512 (0.278)	*ii)* 596 (0.323)
iii) 387 (0.210)	*iv)* 350 (0.190)

$Total_{con}$ = 983 (0. 533)
$Total_{opp}$ = 862 (0. 467)

Fig S8: All SB gains and losses for genes with A,B) large and C,D) small expression changes between the focal and ancestral species in the A,C) head and B,D) body. The cutoff for a large expression change was a $\log_2$ fold-change of magnitude 1 or higher in the head and 2 or higher in the body. Genes with concordant (con) changes in expression between the sexes are located in quadrants ii and iii; while genes with opposing (opp) expression changes between the sexes are located in quadrants i and iv.

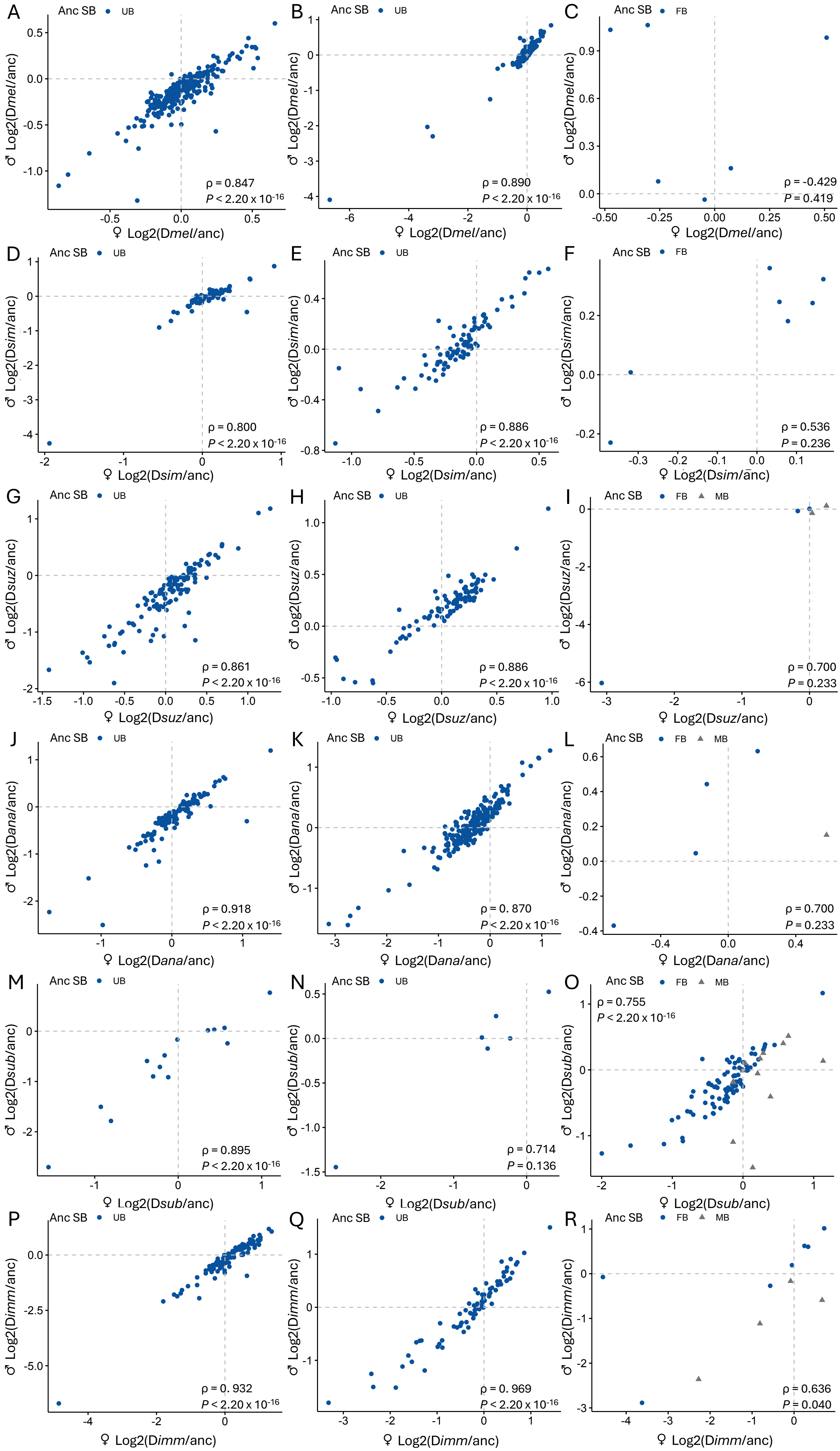
A
Anc SB UB
♂ Log2(Dmel/anc)
♀ Log2(Dmel/anc)
ρ = 0.847
P < 2.20 x 10-16
B
Anc SB UB
♂ Log2(Dmel/anc)
♀ Log2(Dmel/anc)
ρ = 0.890
P < 2.20 x 10-16
C
Anc SB FB
♂ Log2(Dmel/anc)
♀ Log2(Dmel/anc)
ρ = -0.429
P = 0.419
D
Anc SB UB
♂ Log2(Dsim/anc)
♀ Log2(Dsim/anc)
ρ = 0.800
P < 2.20 x 10-16
E
Anc SB UB
♂ Log2(Dsim/anc)
♀ Log2(Dsim/anc)
ρ = 0.886
P < 2.20 x 10-16
F
Anc SB FB
♂ Log2(Dsim/anc)
♀ Log2(Dsim/anc)
ρ = 0.536
P = 0.236
G
Anc SB UB
♂ Log2(Dsuz/anc)
♀ Log2(Dsuz/anc)
ρ = 0.861
P < 2.20 x 10-16
H
Anc SB UB
♂ Log2(Dsuz/anc)
♀ Log2(Dsuz/anc)
ρ = 0.886
P < 2.20 x 10-16
I
Anc SB FB MB
♂ Log2(Dsuz/anc)
♀ Log2(Dsuz/anc)
ρ = 0.700
P = 0.233
J
Anc SB UB
♂ Log2(Dana/anc)
♀ Log2(Dana/anc)
ρ = 0.918
P < 2.20 x 10-16
K
Anc SB UB
♂ Log2(Dana/anc)
♀ Log2(Dana/anc)
ρ = 0. 870
P < 2.20 x 10-16
L
Anc SB FB MB
♂ Log2(Dana/anc)
♀ Log2(Dana/anc)
ρ = 0.700
P = 0.233
M
Anc SB UB
♂ Log2(Dsub/anc)
♀ Log2(Dsub/anc)
ρ = 0.895
P < 2.20 x 10-16
N
Anc SB UB
♂ Log2(Dsub/anc)
♀ Log2(Dsub/anc)
ρ = 0.714
P = 0.136
O
Anc SB FB MB
ρ = 0.755
P < 2.20 x 10-16
♂ Log2(Dsub/anc)
♀ Log2(Dsub/anc)
P
Anc SB UB
♂ Log2(Dimm/anc)
♀ Log2(Dimm/anc)
ρ = 0. 932
P < 2.20 x 10-16
Q
Anc SB UB
♂ Log2(Dimm/anc)
♀ Log2(Dimm/anc)
ρ = 0. 969
P < 2.20 x 10-16
R
Anc SB FB MB
♂ Log2(Dimm/anc)
♀ Log2(Dimm/anc)
ρ = 0.636
P = 0.040

Fig S9: Expression changes during sex bias turnover in the head. Shown are scatterplots of expression in A–C) *Dmel*, D–F) *Dsim*, G–I) *Dsuz*, J–L) *Dana*, M–O) *Dsub*, and P–R) *Dimm* in each sex relative to ancestral (anc) expression for A,D,G,J,M,P) FB or B,E,H,K,N,O) MB gains or C,F,I, L,O,R) losses. Spearman's ρ and the associated *P* value for each category are shown.

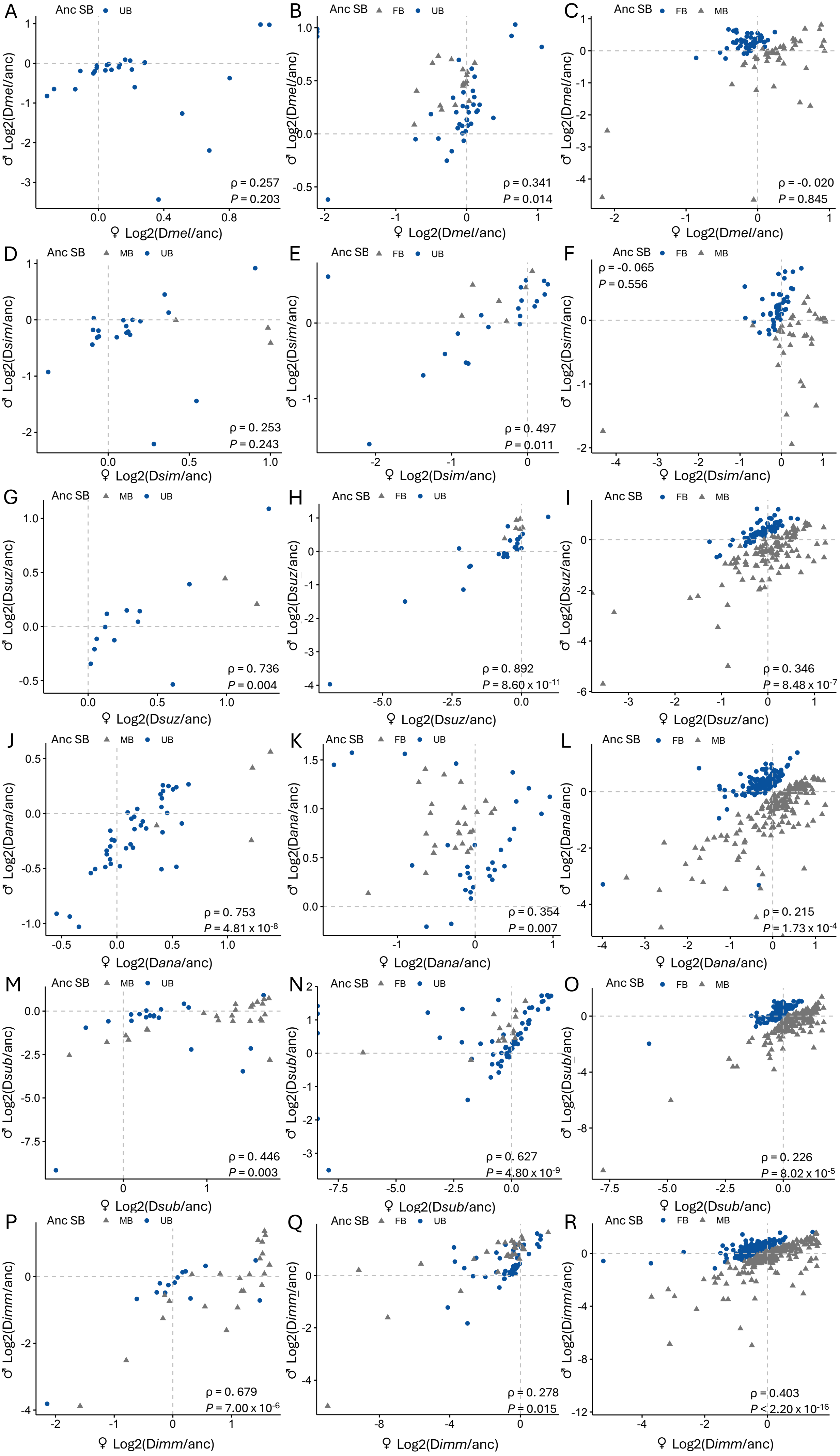
A
Anc SB UB
♂ Log2(Dmel/anc)
♀ Log2(Dmel/anc)
ρ = 0.257
P = 0.203
B
Anc SB FB UB
♂ Log2(Dmel/anc)
♀ Log2(Dmel/anc)
ρ = 0.341
P = 0.014
C
Anc SB FB MB
♂ Log2(Dmel/anc)
♀ Log2(Dmel/anc)
ρ = -0. 020
P = 0.845
D
Anc SB MB UB
♂ Log2(Dsim/anc)
♀ Log2(Dsim/anc)
ρ = 0. 253
P = 0.243
E
Anc SB FB UB
♂ Log2(Dsim/anc)
♀ Log2(Dsim/anc)
ρ = 0. 497
P = 0.011
F
Anc SB FB MB
ρ = -0. 065
P = 0.556
♂ Log2(Dsim/anc)
♀ Log2(Dsim/anc)
G
Anc SB MB UB
♂ Log2(Dsuz/anc)
♀ Log2(Dsuz/anc)
ρ = 0. 736
P = 0.004
H
Anc SB FB UB
♂ Log2(Dsuz/anc)
♀ Log2(Dsuz/anc)
ρ = 0. 892
P = 8.60 x 10-11
I
Anc SB FB MB
♂ Log2(Dsuz/anc)
♀ Log2(Dsuz/anc)
ρ = 0. 346
P = 8.48 x 10-7
J
Anc SB MB UB
♂ Log2(Dana/anc)
♀ Log2(Dana/anc)
ρ = 0. 753
P = 4.81 x 10-8
K
Anc SB FB UB
♂ Log2(Dana/anc)
♀ Log2(Dana/anc)
ρ = 0. 354
P = 0.007
L
Anc SB FB MB
♂ Log2(Dana/anc)
♀ Log2(Dana/anc)
ρ = 0. 215
P = 1.73 x 10-4
M
Anc SB MB UB
♂ Log2(Dsub/anc)
♀ Log2(Dsub/anc)
ρ = 0. 446
P = 0.003
N
Anc SB FB UB
♂ Log2(Dsub/anc)
♀ Log2(Dsub/anc)
ρ = 0. 627
P = 4.80 x 10-9
O
Anc SB FB MB
♂ Log2(Dsub/anc)
♀ Log2(Dsub/anc)
ρ = 0. 226
P = 8.02 x 10-5
P
Anc SB MB UB
♂ Log2(Dimm/anc)
♀ Log2(Dimm/anc)
ρ = 0. 679
P = 7.00 x 10-6
Q
Anc SB FB UB
♂ Log2(Dimm/anc)
♀ Log2(Dimm/anc)
ρ = 0. 278
P = 0.015
R
Anc SB FB MB
♂ Log2(Dimm/anc)
♀ Log2(Dimm/anc)
ρ = 0.403
P < 2.20 x 10-16

Fig S10: Expression changes during sex bias turnover in the body. Shown are scatterplots of expression in A–C) *Dmel*, D–F) *Dsim*, G–I) *Dsuz*, J–L) *Dana*, M–O) *Dsub*, and P–R) *Dimm* in each sex relative to ancestral (anc) expression for A,D,G,J,M,P) FB or B,E,H,K,N,O) MB gains or C,F,I, L,O,R) losses. Spearman's ρ and the associated *P* value for each category are shown.

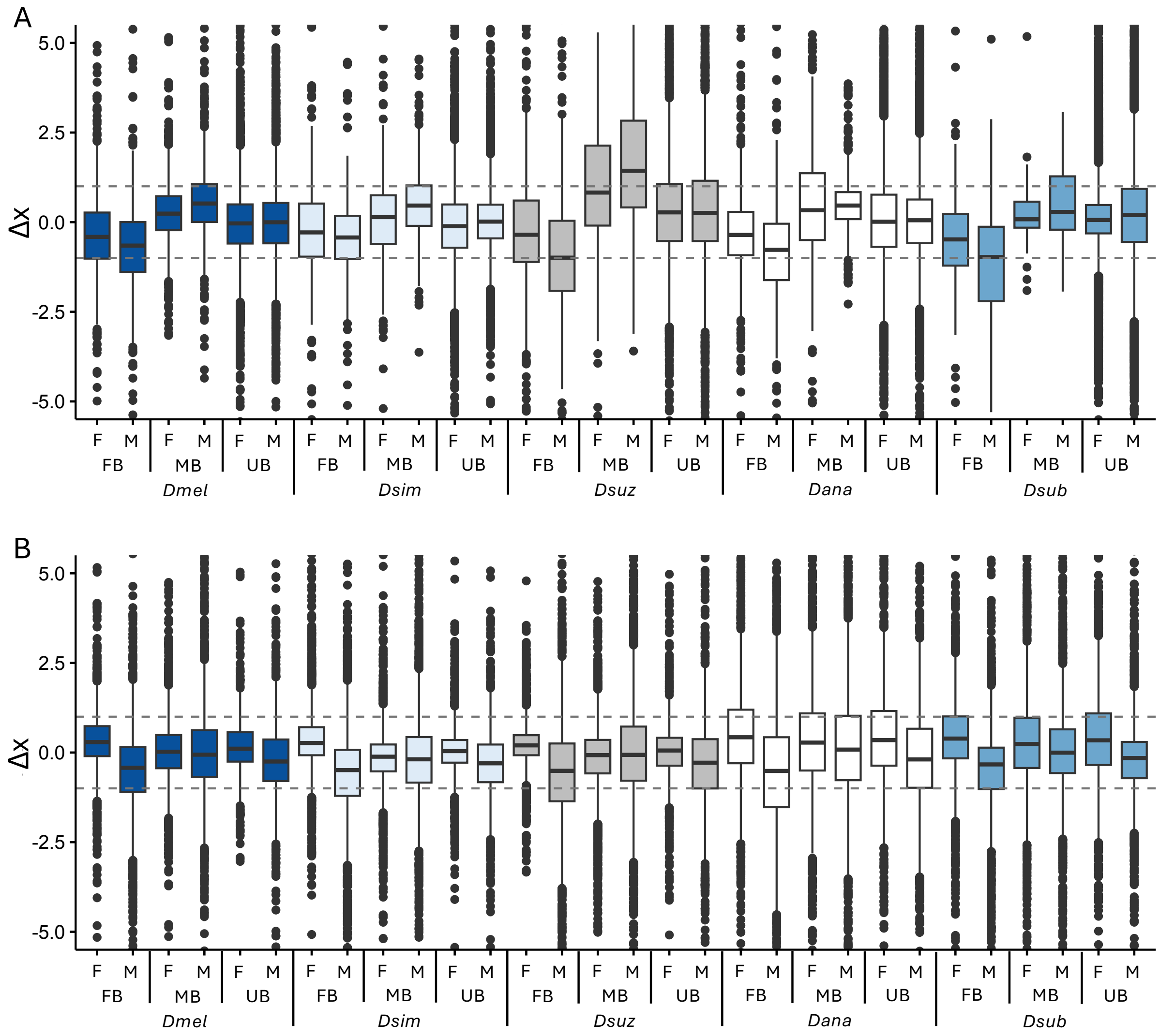

Fig S11: Δx in the A) head and B) body of 5 *Drosophila* species. Shown are Δx values for FB, MB and UB genes in females (F) and males (M) of *Dmel*, *Dsim*, *Dsuz*, *Dana*, and *Dsub*. The dashed gray line represents the cutoff above or below which a gene shows signs of putative positive selection. For better visualization, Δx values above 5 or below -5 are not shown.

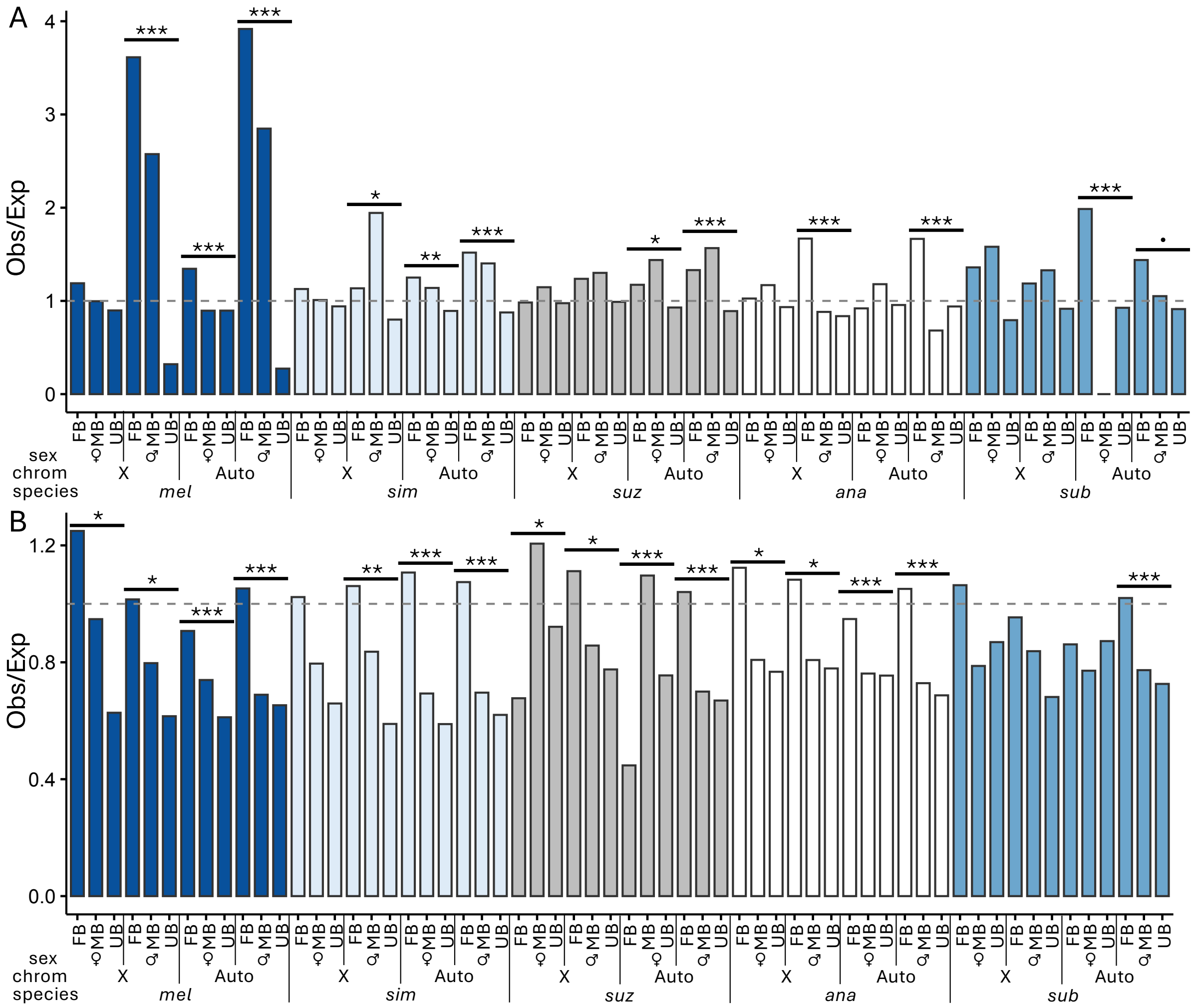

Fig S12: Chromosomal distribution of signs of putative positive selection at the gene expression level. Shown are the ratios of observed (obs) to expected (exp) number of genes under putative positive selection ($\Delta x > 1$ or < -1) in each sex bias category and sex on the X chromosome and autosomes (auto) in the A) head and B) body. Significance was assessed with a χ^2 test. ****P* < 0.005, ***P* < 0.01, **P* < 0.05, marginally non-significant: .*P* < 0.1. Non-significant comparisons not shown.

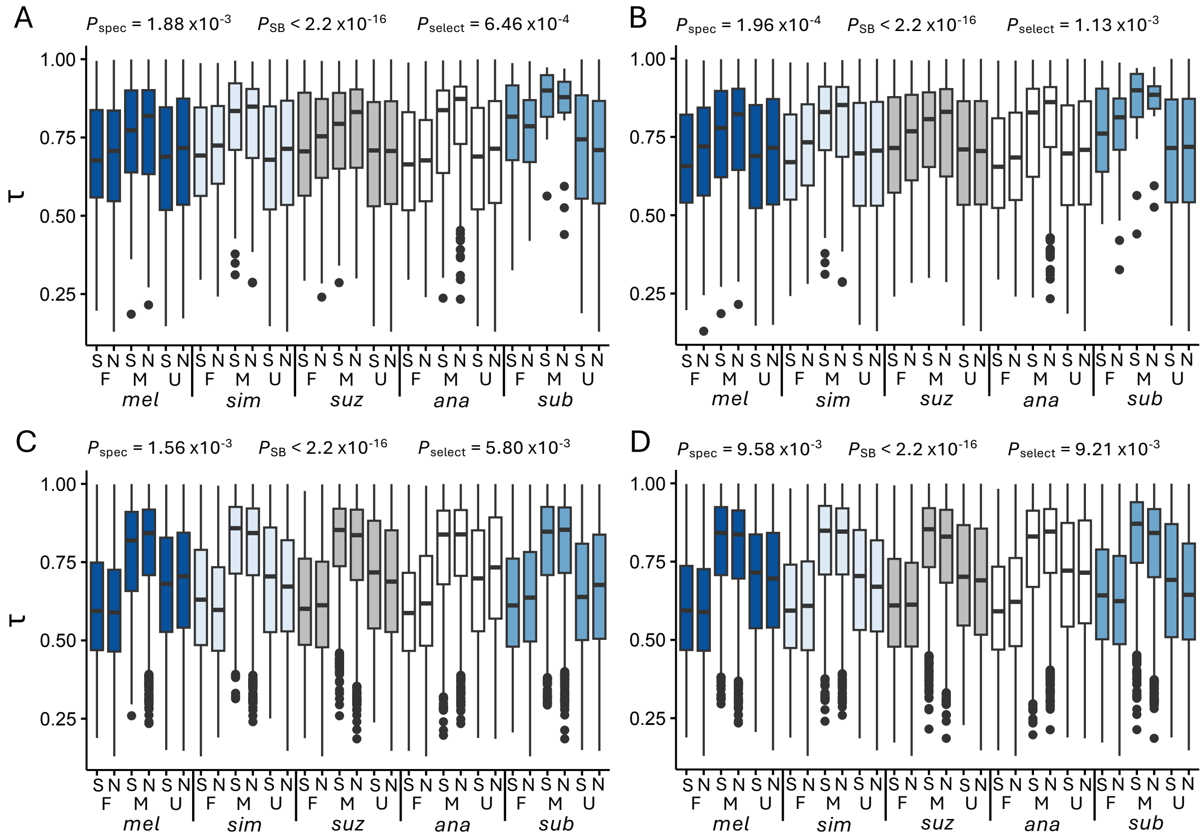

Fig S13: Tissue specificity, τ, in genes identified as putatively under positive selection (S) or not under selection (N) in A,C) females (F) and B,D) males (M) the A,B) head and C,D) body for MB (M), FB (F), and UB (U) genes in *Dmel*, *Dsim*, *Dsuz*, *Dana*, *Dsub*, and *Dimm*. Significance was assessed for each body part with a type II ANOVA with sex bias (SB) category, species, and if under putative positive selection as factors.

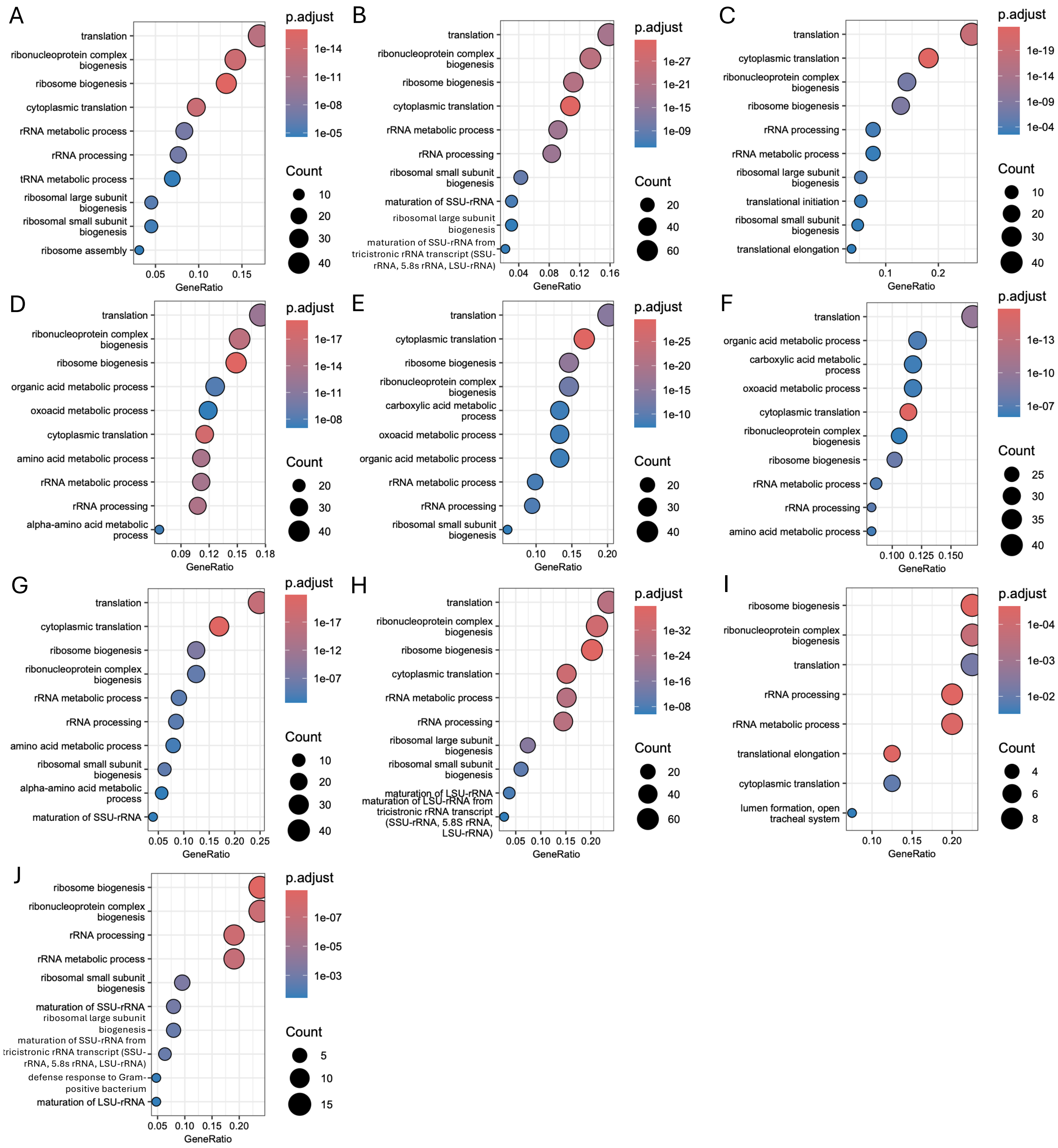

Fig S14: Dotplots showing the 10 most enriched biological processes for FB genes detected as A,C,E,G,I) putatively positively selected or B,D,F,H,J) non-selected in females in A,B) *Dmel*, C,D) *Dsim*, E,F) *Dsuz*, G,H) *Dana*, or I,J) *Dsub*. Colors indicate the BH-corrected P-value, while dot size indicates the number of genes in a GO category.

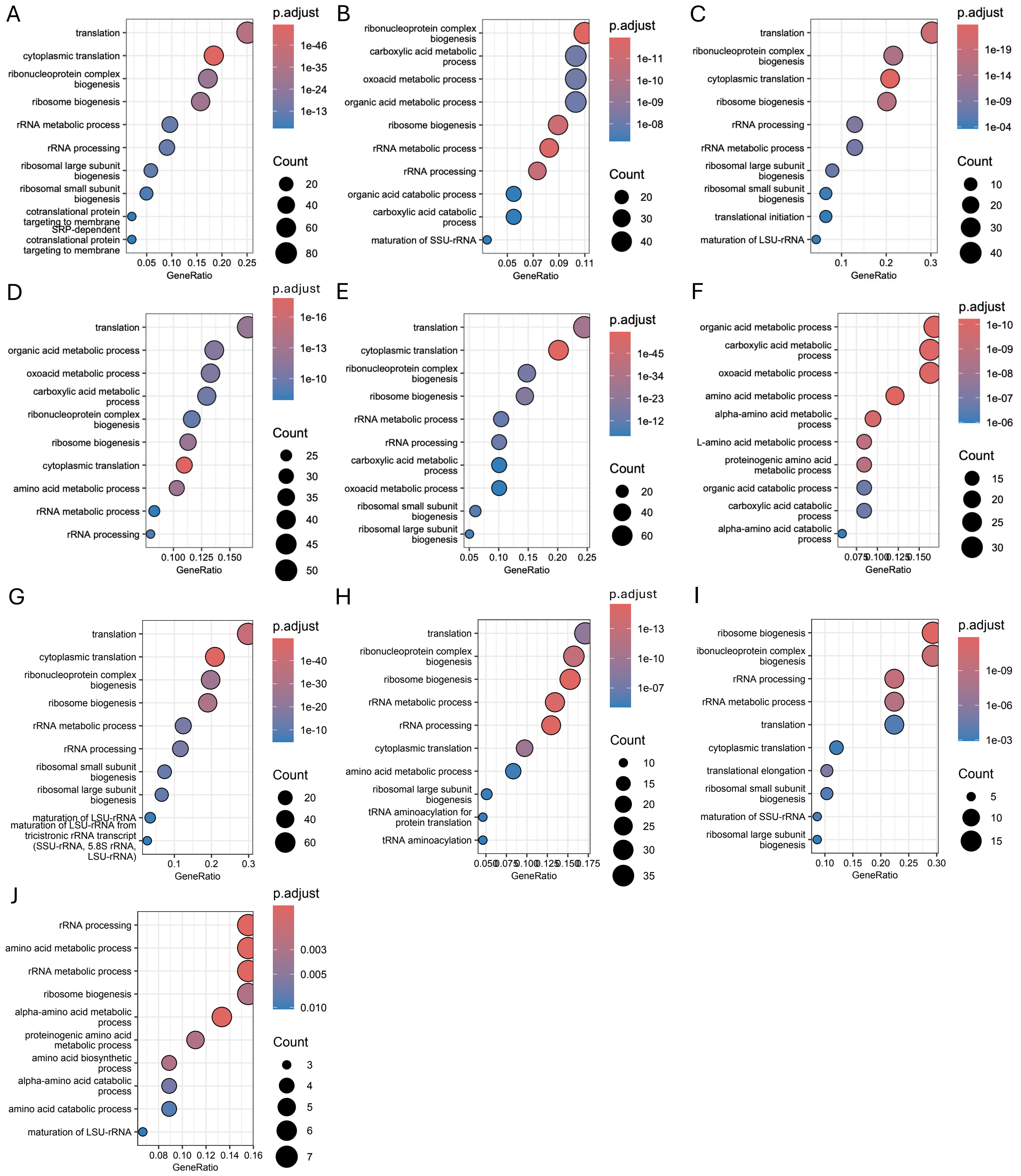

Fig S15: Dotplots showing the 10 most enriched biological processes for FB genes detected as A,C,E,G,I) putatively positively selected or B,D,F,H,J) non-selected in males in A,B) *Dmel*, C,D) *Dsim*, E,F) *Dsuz*, G,H) *Dana*, or I,J) *Dsub*. Colors indicate the BH-corrected P-value, while dot size indicates the number of genes in a GO category.

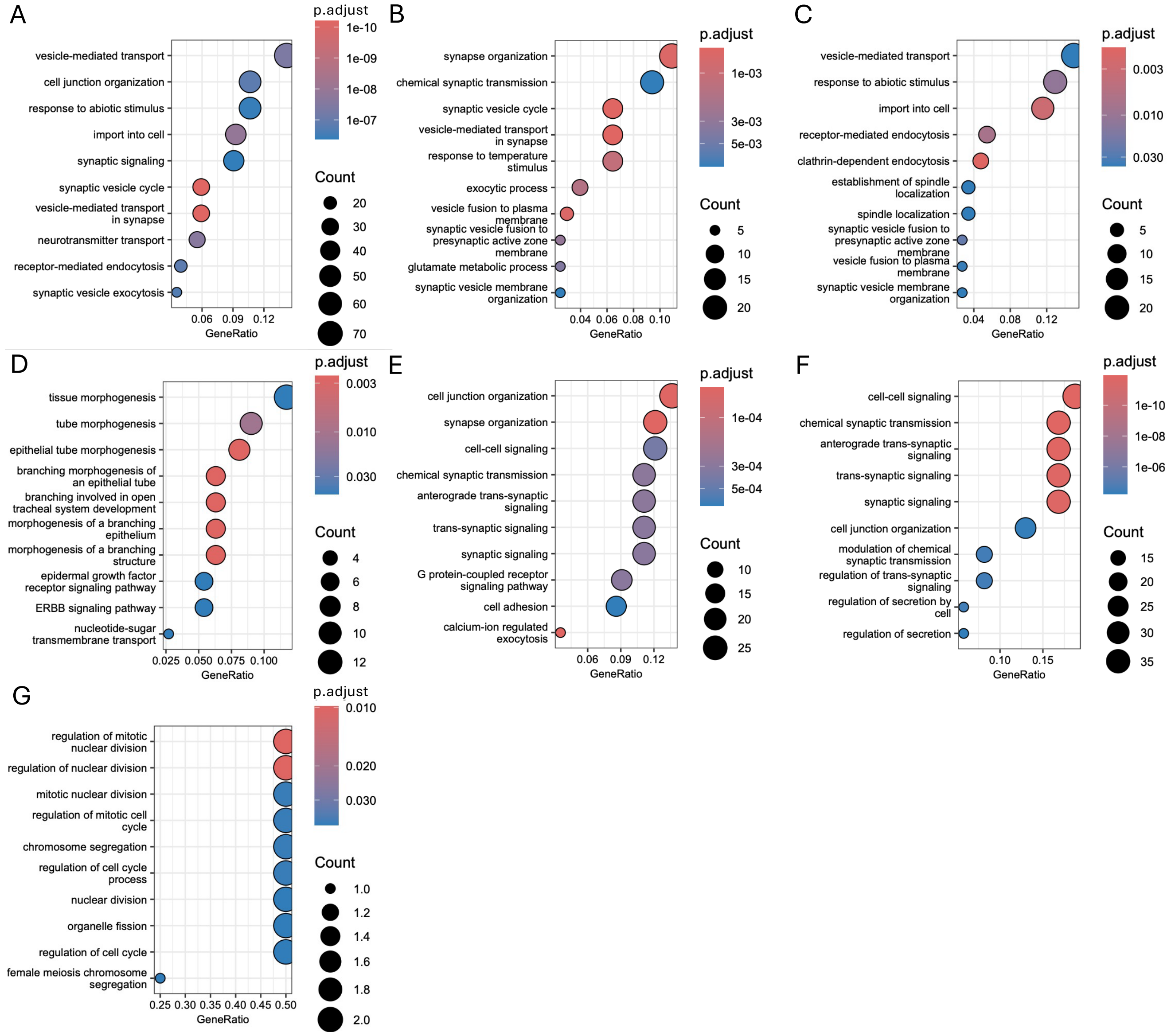

Fig S16: Dotplots showing the 10 most enriched biological processes for MB genes detected as C,E,G) putatively positively selected or A,B,D,F) non-selected in females in A) *Dmel*, B) *Dsim*, C,D) *Dsuz*, E,F) *Dana*, or G) *Dsub*. Colors indicate the BH-corrected P-value, while dot size indicates the number of genes in a GO category. Comparisons with no significant GO enrichment not shown.

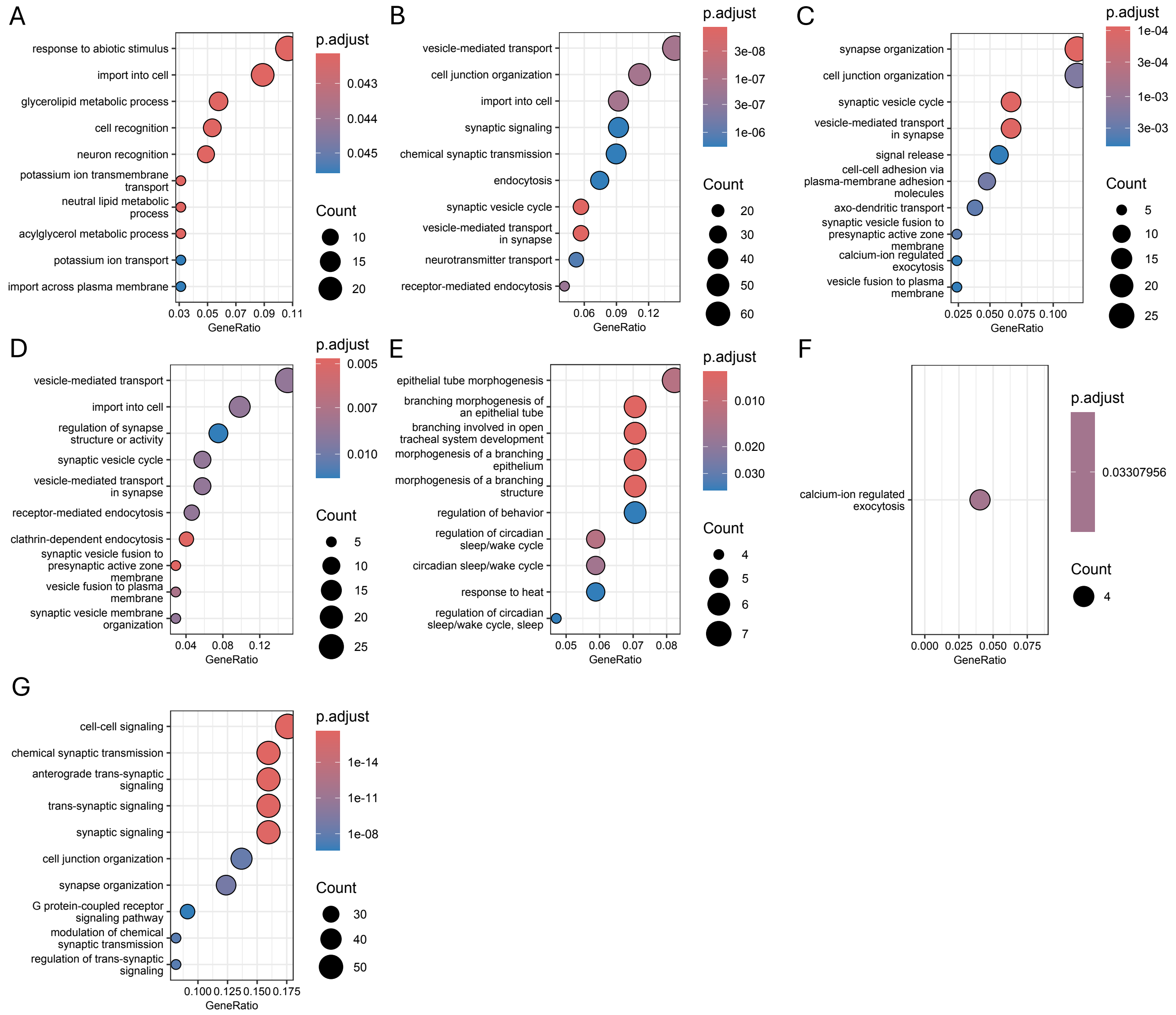

Fig S17: Dotplots showing the 10 most enriched biological processes for MB genes detected as A,D,F) putatively positively selected or B,C,E,G) non-selected in males in A,B) *Dmel*, C) *Dsim*, D,E) *Dsuz*, or F,G) *Dana*. Colors indicate the BH-corrected P-value, while dot size indicates the number of genes in a GO category. Comparisons with no significant GO enrichment not shown.

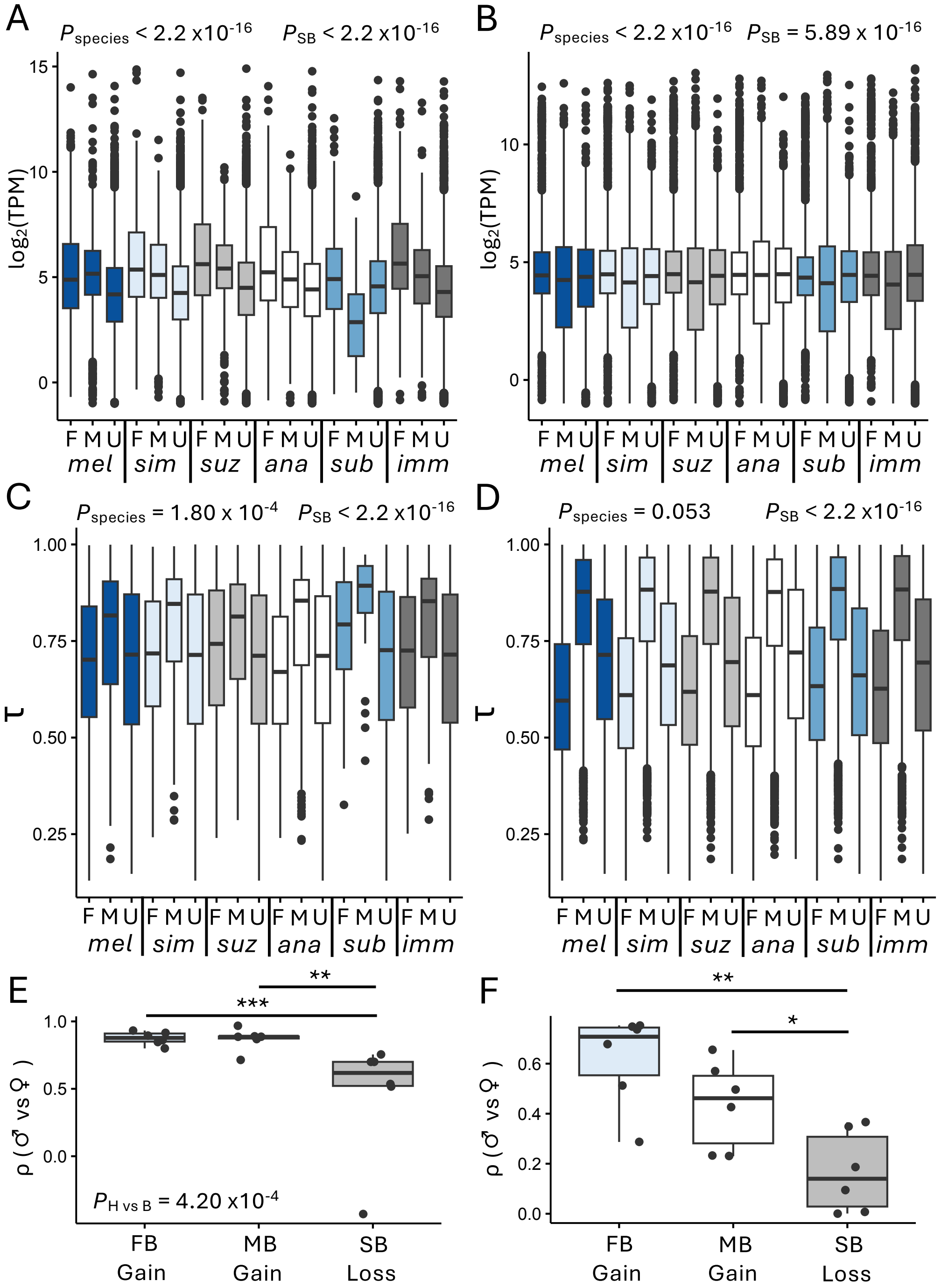

G

	i) 223 (0.129)	ii) 614 (0.356)
	iii) 688 (0.399)	iv) 199 (0.115)

$Total_{con}$ = 1302 (0.755)
$Total_{opp}$ = 422 (0.245)

H

	i) 514 (0.284)	ii) 579 (0.320)
	iii) 393 (0.217)	iv) 326 (0.180)

$Total_{con}$ = 972 (0. 536)
$Total_{opp}$ = 840 (0. 464)

I

	i) 2 (0.020)	ii) 21 (0.210)
	iii) 74 (0.740)	iv) 3 (0.030)

$Total_{con}$ = 95 (0.950)
$Total_{opp}$ = 5 (0.050)

J

	i) 11 (0.212)	ii) 2 (0. 038)
	iii) 35 (0. 673)	iv) 4 (0.077)

$Total_{con}$ = 37 (0. 712)
$Total_{opp}$ = 15 (0. 288)

K

	i) 221 (0.136)	ii) 593 (0.365)
	iii) 614 (0.378)	iv) 196 (0.121)

$Total_{con}$ = 1207 (0.743)
$Total_{opp}$ = 417 (0.257)

L

	i) 503 (0.286)	ii) 577 (0.328)
	iii) 358 (0.203)	iv) 322 (0.183)

$Total_{con}$ = 935 (0. 531)
$Total_{opp}$ = 825 (0. 469)

Fig S18: Expression analyses using a TPM cut-off of 0.5. A–D) Shown are A,B) overall expression levels and C,D) tissue specificity τ in the A,C) head and B,D) body for MB (M), FB (F), and UB (U) genes in *Dmel* (*mel*), *Dsim* (*sim*), *Dsuz* (*suz*), *Dana* (*ana*), *Dsub* (*sub*), and *Dimm* (*imm*). A,B) Significance was assessed for each body part with a type II ANOVA with sex bias (SB) category and species as fixed factors and gene as a random factor. C,D) Significance was assessed for each body part with a type II ANOVA with SB category and species as factors. E,F) Shown are Spearman's ρ correlations between male and female expression changes within each species for SB gains and losses in the E) head (H) and F) body (B). Significance was assessed with a Mann-Whitney U test. ***P < 0.005, **P < 0.01, *P < 0.05. Non-significant comparisons not shown. G–L) Genes with concordant (con) changes in expression between the sexes are located in quadrants ii and iii; while genes with opposing (opp) expression changes between the sexes are located in quadrants i and iv. Total genes in each quadrant for the entire phylogeny in the G) head and H) body are shown. In parentheses, the proportion of total gene expression changes are shown. G–J) Shown are all SB gains and losses for genes with I,J) large and K,L) small expression changes between the focal and ancestral species in the I,K) head and J,L) body. The cut-off for a large expression change was a $\log_2$ fold-change of magnitude 1 or higher in the head and 2 or higher in the body.

Supplementary Data Legends

Data S1: Data underlying gene expression analyses: gene counts.

Data S2: Data underlying gene expression analyses: TPM.

Data S3: Data underlying gene expression analyses: SB gene expression, τ, and expression changes.

Data S4: Data underlying selection analyses.
